# A Facile Fluorometric Assay of Orotate Phosphoribosyltransferase Activity Using a Selective Fluorogenic Reaction for Orotic Acid

**DOI:** 10.3390/s23052507

**Published:** 2023-02-24

**Authors:** Takayuki Shibata, Tomohiro Narita, Yutaka Suto, Hasina Yasmin, Tsutomu Kabashima

**Affiliations:** 1 Department of Laboratory Sciences, Gunma University Graduate School of Health Sciences, 3-39-22 Showa-machi, Maebashi 371-8514, Japan; 2 Department of Pharmaceutical Sciences, Nagasaki University, 1-14 Bunkyo-machi, Nagasaki 852-8521, Japan; 3 Faculty of Pharmacy, Takasaki University of Health and Welfare, 37-1 Nakaoruimachi, Takasaki 370-0033, Japan; 4 Department of Pharmacy, BRAC University, 66 Mohakhali, Dhaka 1212, Bangladesh; 5 Graduate School of Pharmaceutical Sciences, Nagasaki International University, 2825-7 Huis Ten Bosch, Sasebo 859-3298, Japan

**Keywords:** orotic acid, fluorogenic reaction, orotate phosphoribosyltransferase, enzyme activity, laboratory test

## Abstract

Orotate phosphoribosyltransferase (OPRT) exists as a bifunctional enzyme, uridine 5′-monophosphate synthase, in mammalian cells and plays an important role in pyrimidine biosynthesis. Measuring OPRT activity has been considered important for understanding biological events and development of molecular-targeting drugs. In this study, we demonstrate a novel fluorescence method for measuring OPRT activity in living cells. The technique utilizes 4-trifluoromethylbenzamidoxime (4-TFMBAO) as a fluorogenic reagent, which produces selective fluorescence for orotic acid. To perform the OPRT reaction, orotic acid was added to HeLa cell lysate, and a portion of the enzyme reaction mixture was heated at 80 °C for 4 min in the presence of 4-TFMBAO under basic conditions. The resulting fluorescence was measured using a spectrofluorometer, which reflects the consumption of orotic acid by the OPRT. After optimization of the reaction conditions, the OPRT activity was successfully determined in 15 min of enzyme reaction time without further procedures such as purification of OPRT or deproteination for the analysis. The activity obtained was compatible with the value measured by the radiometric method with [^3^H]-5-FU as the substrate. The present method provides a reliable and facile measurement of OPRT activity and could be useful for a variety of research fields targeting pyrimidine metabolism.

## 1. Introduction

**Figure 1:**
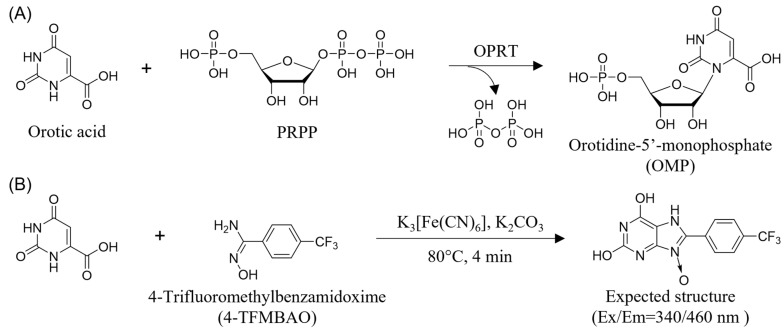
(**A**) The biosynthetic reaction from orotic acid and PRPP to OMP catalyzed by OPRT. (**B**) The fluorogenic reaction specific for orotic acid using 4-TFMBAO.

In the de novo synthesis of nucleic acids, pyrimidine and purine nucleotides are synthesized via different pathways. Unlike purine bases, pyrimidine nucleotides are synthesized by condensation of the nucleobase moiety and phosphorylated ribose. The first step in this route is the condensation of 5-phosphoribosyl-1-pyrophosphate (PRPP) with orotic acid, which is catalyzed by orotate phosphoribosyltransferase (OPRT) (Figure 1A) [1,2]. The resulting orotidine-5′-monophosphate (OMP) is subsequently decarboxylated to uridine 5′-monophosphate (UMP) by orotidine-5′-monophosphate decarboxylase (ODC), and UMP is converted to other pyrimidine nucleotides such as cytidine 5′-monophosphate (CMP) and thymidine 5′-monophosphate (TMP). In humans, OPRT, along with ODC, is present as part of the bifunctional enzyme UMP synthase (UMPS), and the loss of even one of these activities results in the interception of pyrimidine biosynthesis [3]. Thus, OPRT plays an important role in nucleic acid metabolism and is a target for various biological events.

5-Fluorouracil is an anticancer agent used in a wide range of cancers [4,5]. Various analogs and prodrugs have been developed and used clinically [6,7]. These fluoropyrimidine anticancer agents exert their effects by inhibiting nucleic acid metabolism. The administered fluoropyrimidines are incorporated into PRPP by OPRT and converted to 5-fluoroorotidine-5′-monophosphate (FOMP), which is then converted to 5-fluoro-UMP by UMPS [8]. 5-Fluoro-UMP is finally converted to 5-fluoro-UTP and 5-fluoro-deoxyUTP, which cause metabolic disorders, such as RNA transcription inhibition and DNA replication inhibition. Oteracil is a selective OPRT inhibitor and is often administered concurrently with fluoropyrimidines to reduce the gastrointestinal toxicity of 5-FU [9]. Furthermore, it has been reported that the decreased OPRT activity in tumor cells may cause resistance to chemotherapy with 5-FU [8,10]

Orotic aciduria is a pathologic condition characterized by elevated concentrations of orotic acid and orotidic acid in urine. This symptom is known to be caused by the deactivation of UMPS and urea cycle inhibition such as transcarbamylase deficiency [11]. In particular, UMPS deficiency includes the loss of OPRT activity, resulting in decreased production of UMP [12]. This inborn error causes dysfunction of myeloid and neuronal cells due to inhibition of DNA/RNA synthesis, leading to serious diseases such as megaloblastic anemia, microcytic anemia and mental retardation. In parasitology, OPRT has received increasing attention. For example, parasitic trypanosomatids such as *Trypanosoma brucei* possess enzymes required for both de novo biosynthesis and purine nucleotide salvage. However, in vivo pyrimidine salvage in *Trypanosoma brucei* is insufficient, and knocking out the UMPS gene in mice causes depletion of pyrimidine nucleotides in the parasite [13,14]. Malaria parasites also lack the salvage pathway of pyrimidines and depend on de novo synthesis to supply pyrimidine nucleotides [15]. These results suggest that OPRT could be an important target for antiparasitic therapies.

Therefore, the measurement of OPRT activity in living cells is important for numerous research fields such as biochemical studies, drug discovery and laboratory testing. The conventional methods utilized radiometric techniques using radiolabeled orotic acid [16] and 5-FU [17]. Because the use of radioactive substrates is discouraged, alternative non-radiometric methods, such as high-performance liquid chromatography (HPLC) [18] and spectrometric methods [15], have been developed. However, the HPLC method requires a long assay time and is not suitable for the measurement of a large number of specimens. Spectrometric methods require purification of the enzyme before assaying the biological samples. Therefore, high-throughput assay methods are in high demand.

Recently, we have developed a novel fluorescent reaction that provides rapid and specific fluorogenic derivatization of orotic acid using 4-trifluoromethylbenzamidoxime (4-TFMBAO) (Figure 1B) [19,20]. The reaction requires heating at 80 °C for several minutes in the presence of K_3_[Fe(CN)_6_] and K_2_CO_3_, and the orotic acid can be selectively converted to its fluorescence (FL) derivative. The reaction has also been applied to the measurement of orotic acid in biological samples, which includes quantification of orotic acid in urine [19] and determination of the activity of an enzyme that produces orotic acid as a product [20]. In this study, we applied this method to assay OPRT activity in cultured cells. Since OPRT consumes orotic acid as a substrate, the decrease in FL intensity corresponding to the consumption of orotic acid should be traced. We demonstrated that OPRT activity can be obtained by quantifying the decrease in orotic acid by 4-TFMBAO reaction, and the present method affords a facile analysis of OPRT activity in biological samples without laborious procedures such as the use of radiolabeled substrate and purification of OPRT.

## 2. Materials and Methods

### 2.1. Chemicals and Materials

Orotic acid (OA) and 5-fluoroorotic acid (FOA) were purchased from TCI (Kyoto, Japan). 5-Phosphoribosyl-1-pyrophosphate (PRPP) and 5-fluoroorotic acid were obtained from Santa Cruz Biotechnology, Inc. (Dallas, TX, USA). 4-Trifluoromethylbenzamidoxime (4-TFMBAO) was purchased from Sigma-Aldrich (Saint Louis, MO, USA). Other chemicals were of analytical or guaranteed reagent grade. All the materials were used without further purification. The H_2_O used for the experiments was purified using a Milli-Q system.

### 2.2. Cell Culturing

HeLa cells were cultured in 10 cm dishes in Dulbecco’s modified eagle medium containing 10% Fetal Bovine Serum (FBS), 100 units/mL penicillin, 0.1 mg/mL streptomycin and 0.25 μg/mL amphotericin B. Cells were removed from the dish by trypsin when they reached 90% confluence. After washing with 1× PBS, the cells were stored at −80 °C before analysis.

### 2.3. Preparation of Cell Lysate

The cells were thawed in 400 μL of a buffer consisting of 25 mM KH_2_PO_4_-K_2_HPO_4_ (pH 8.3) and 1.25 mM Dithiothreitol (DTT), then sonicated at the maximal output for 4 cycles of 30 s at intervals of 30 s using a BioLogics Ultrasonic Homogenizer Model 300 V/T (Manassas, VA, USA). The lysates were then centrifuged at 12,000× *g* for 20 min, and the supernatants were collected. Protein concentration was measured using the Quick StartTM Bradford 1× Dye Reagent (Bio-Rad Laboratories, Inc., Hercules, CA, USA).

### 2.4. Condition of Enzyme Reaction for OPRT

Unless otherwise specified, each lysate containing 200 μg protein was incubated with final concentrations of 10 mM KH_2_PO_4_-K_2_HPO_4_ (pH8.3), 0.5 mM DTT, 4 mM MgCl_2_, 0.1–1 mM PRPP and 2.5–10 μM orotic acid in a total volume of 500 μL of enzymatic reaction mixture. The enzymatic reaction was performed at 37 °C for 60 min. Statistical analysis of the data was conducted on a computer using JMP Pro 15 software (SAS Institute, Cary, NC, USA).

### 2.5. FL Reaction and Measurement

The enzyme activity of OPRT was measured by detecting a decrease in orotic acid using the fluorogenic derivatization with 4-TFMBAO. Portions (80 μL) of the reaction mixture taken 15, 30 and 60 min after beginning the enzymatic reaction were mixed with 120 μL of H_2_O, 200 μL of 4 mM 4-TFMBAO, 200 μL of 8 mM K_3_[Fe(CN)_6_] and 200 μL of 80 mM K_2_CO_3_ in H_2_O. The mixtures were immediately heated at 80 °C for 4 min and then cooled in an ice bath for 2 min or longer to stop the reaction. The fluorescent intensity was measured on an FP-8300 spectrofluorometer (Jasco, Tokyo, Japan) at excitation and emission wavelengths of 340 nm and 460 nm, respectively.

## 3. Results and Discussion

### 3.1. Principle of the Present Method

**Figure 2:**
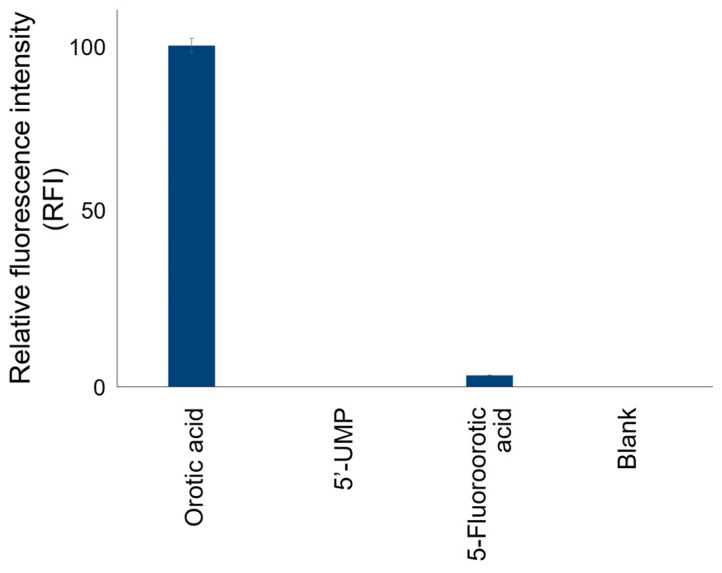
The reaction specificity of the present fluorogenic reaction with 4-TFMBAO to substances associated with OPRT reaction.

The fluorogenic reaction with 4-TFMBAO is highly specific for orotic acid and does not produce FL with most biological materials, including other nucleobases, nucleotides, sugars, amino acids and metabolic small molecules (Supplementary Figure S1). We also found that the reaction was FL-inactive to 5,6-dihydroorotic acid, the precursor of orotic acid, and UMP, the product of UMPS (Figure 2). Since OMP synthesized from orotic acid by OPRT activity is directly converted to UMP by ODC, the activity of OPRT could be calculated by quantifying the decrease in orotic acid added as the substrate of UMPS.

### 3.2. Detection of OPRT Activity by the Fluorogenic Reaction with 4-TFMBAO

**Figure 3:**
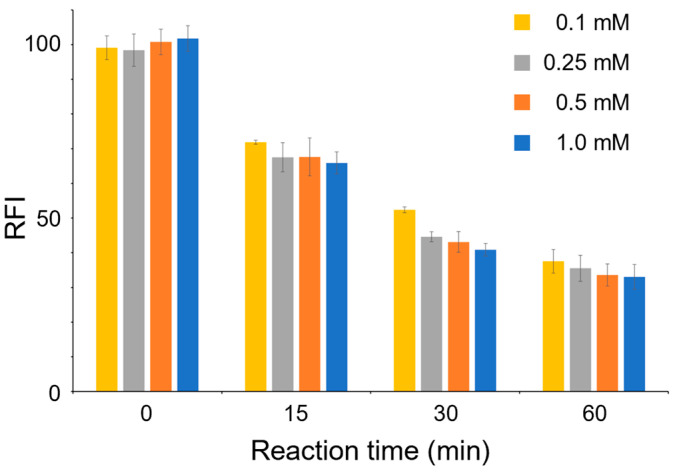
The effect of PRPP concentration on fluorescence intensity obtained after OPRT reaction followed by the fluorogenic reaction with 4-TFMBAO. All experiments were repeated three times on different days.

The effect of PRPP concentration on the OPRT reaction was first evaluated. To the HeLa cell lysate, corresponding to 200 μg of total protein, orotic acid was added and the mixture was incubated with different concentrations of PRPP (0.25, 0.5 and 1.0 mM). A portion of the reaction solution was collected at reaction times of 0, 15, 30 and 60 min, and a fluorogenic reaction was performed to measure the fluorescence intensity at Ex/Em = 340/460 nm. At all PRPP concentrations, the fluorescence intensity derived from orotic acid decreased with reaction time. These results suggested that the OPRT reaction proceeds under these reaction conditions and the orotic acid consumed by OPRT could be pursued using the present method. A PRPP concentration of 250 μM was used in a previous study to measure yeast OPRT activity [16]. In the present study using mammalian cells, the velocity of OPRT activity did not reach a plateau even at 500 μM PRPP (Figure 3). At 30 min of OPRT reaction, the relative fluorescence intensities (RFI) with 0.5 mM and 1.0 mM PRPP were 43.11 ± 2.99 and 40.84 ± 1.82, respectively. Although these mean values were slightly different, these error bars overlapped. At 60 min of OPRT reaction, RFI with 0.5 mM and 1.0 mM PRPP were 33.60 ± 3.19 and 33.05 ± 3.54, respectively, and these values as well as standard deviation values were almost the same. These results suggested that OPRT reaction was saturated with 1.0 mM PRPP, and this concentration was selected as the optimum condition to obtain the maximum OPRT activity.

### 3.3. Optimization of the Substrate Concentration

**Figure 4:**
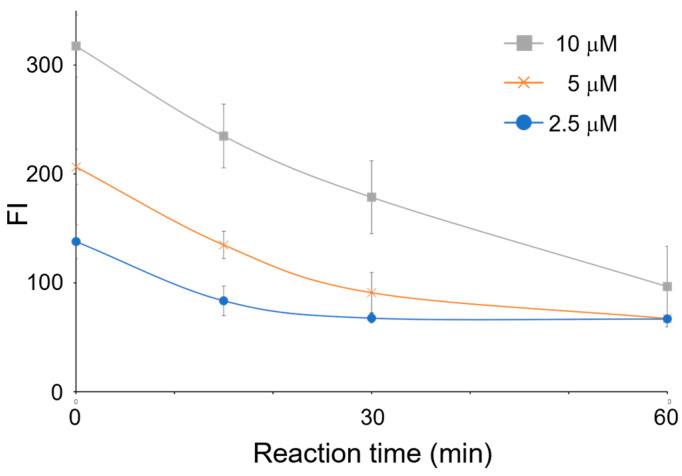
Time-dependent decrease in FL intensity on OPRT reaction. Different concentrations of orotic acid solutions were incubated with HeLa cell lysate, and a portion of the reaction mixture was allowed for the fluorogenic reaction with 4-TFMBAO. All experiments were repeated three times on different days.

In a typical enzyme activity assay, a sufficient amount of substrate is added to obtain maximum enzyme activity. However, the decrease in orotic acid is measured to calculate OPRT activity in this method, and an excess substrate should lead to an increase in the FL signal. The relative difference in the FL signals before and after the reaction should be shrunk, resulting in enlarging an error in activity measurement. To overcome this problem, a minimal amount of substrate concentration that gives the highest enzyme activity should be explored. Figure 4 shows the time-dependent reduction in FL intensity with different concentrations of orotic acid. Good linearity was obtained within 30 min of the enzyme reaction time, and the reaction reached the endpoint at approximately 60 min (Figure 4). The initial rate for 30 min with 5.0 μM orotic acid was larger than that with 2.5 μM orotic acid, whereas 10 μM orotic acid exhibited a similar reaction rate to 5 μM orotic acid. The absolute value of the slope of the approximate curve obtained with 10 μM orotic acid for 30 min (y = −3.37x + 304, R^2^ = 0.997) was slightly larger than that with 5.0 μM orotic acid (y = −3.02x + 194, R^2^ = 0.998). In order to further investigate the parallelism of these lines, the datasets obtained from OPRT reaction for 30 min were statistically analyzed. The analysis was conducted using analysis of covariance model with the null hypothesis of no interaction between orotic acid concentration and OPRT reaction time (Supplementary Figure S2). When parallelism of all three lines were analyzed, the resulting *p*-value was calculated to be 0.032. On the other hand, the *p*-value obtained with two lines, 5 μM and 10 μM orotic acid, was 0.404. In the previous report measuring OPRT activity, the enzyme reaction was conducted for 30 min in the presence of 10 μM radiolabeled substrate [21]. Nevertheless, 15 min reaction time seemed to be enough to obtain a significant difference in FL intensities. We therefore concluded that a reaction time of 15 min with 10 μM orotic acid was sufficient to calculate OPRT activity.

### 3.4. Measurement of OPRT Activity in HeLa Cell

**Figure 5:**
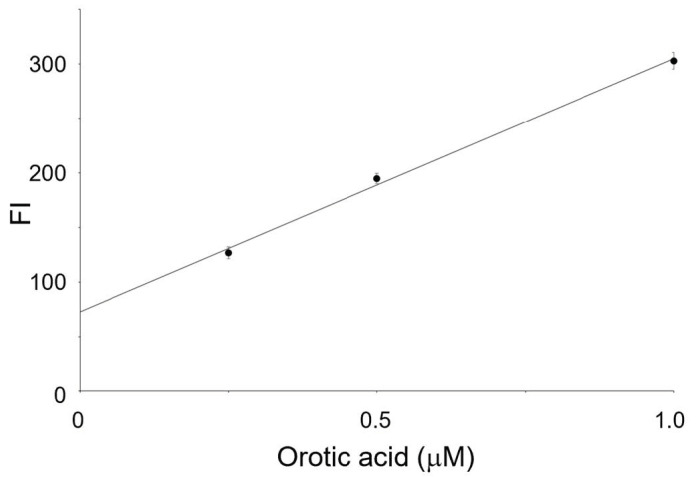
The calibration curve of orotic acid by FL in the presence of the same amount of cell lysate used in the OPRT reaction. All experiments were repeated three times on different days.

A large number of co-existences are included in the cell lysates. Although the 4-TFMBAO reaction is highly specific to orotic acid, their impurities should produce an unignorable FL value. Furthermore, the efficiency of the fluorogenic reaction should be highly affected by these co-existences, which makes it difficult to measure enzyme activity appropriately. Therefore, a calibration curve for orotic acid should be created in the presence of the same amount of cell lysate. In this study, the calibration curve for orotic acid in the specimen was created by quick heating of the mixture after adding an orotic acid of a known concentration to a solution containing fluorogenic reagents and the same amount of lysed cells used in the ORPT reaction. Three independent experiments were conducted on different days to determine the precision of the proposed method. Good linearity between the orotic acid concentration and FL intensity was obtained with a satisfactory standard deviation at each concentration point (y = 232x + 72.7, R^2^ = 0.997) (Figure 5). The OPRT activity of the cultured HeLa cells used in this study was calculated to be 27.7 nmol/h/mg protein. It should be noted that the y-intercept value of the calibration curve was almost the same as the FL intensity at the point where the OPRT consumed the added orotic acid completely (66.8 ± 3.80 for 2.5 μM orotic acid at 60 min and 67.3 ± 3.12 for 5.0 μM orotic acid at 60 min, respectively) (Figure 4). These results clearly indicated that the present method is capable of measuring the amount of orotic acid in biological samples.

### 3.5. Inhibition Assay

**Figure 6:**
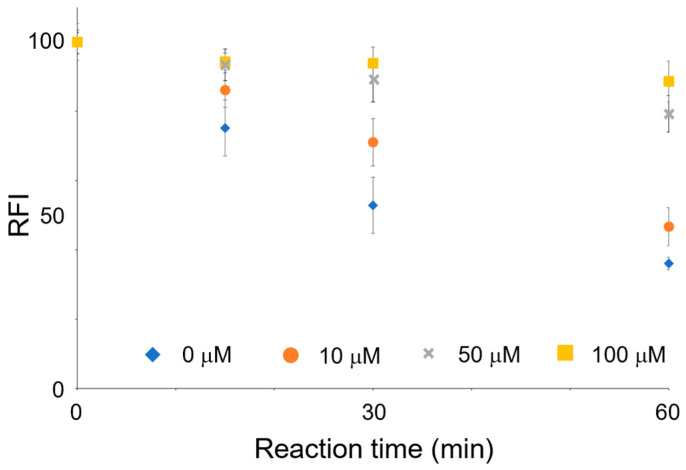
The inhibition effect of 5-fluoroorotic acid on FL production. OPRT activity was restricted by the inhibitor, resulting in diminished consumption of orotic acid. All experiments were repeated three times on different days.

An inhibition experiment targeting OPRT was conducted to verify whether the decrease in FL intensity was derived from the consumption of orotic acid by OPRT. 5-Fluoroorotic acid is a known OPRT inhibitor with high inhibitory activity [22] and is considered to be suitable in this experiment, since 5-fluoroorotic acid did not generate FL with the 4-TFMBAO reaction (Supplementary Figure S3). An aqueous solution of 5-fluoroorotic acid was added to the HeLa cell lysate, and the mixture was pre-incubated prior to the addition of orotic acid. A slight decrease in the FL intensity was observed when the same amount of 5-fluoroorotic acid was used as the substrate (Figure 6), indicating that the OPRT reaction was interrupted and the consumption of orotic acid was reduced. The inhibitory effect became stronger as the concentration of the inhibitor increased, and 10 times more inhibitor than substrate led to near-complete inhibition of OPRT. These results demonstrated that the present method clearly measures OPRT activity.

## 4. Conclusions

In this study, we have developed an FL method for the measurement of OPRT activity in living cells using a fluorogenic reaction selective for orotic acid. The procedures are simple; the mixture of cell lysate and orotic acid was incubated for a maximum of 30 min, followed by heating for 4 min following the addition of fluorogenic reagents. According to the previous report, OPRT activity of four mammarian cancer cell lines ranged from 1 to 59 nmol/h/mg protein [23]. Since the value (27.7 nmol/h/mg) obtained in this study was within this range, the current method could allow reasonable measurement of OPRT activity.

The assay requires only typical equipment such as a sonicator for cell lysate, a dry block heater for the enzyme reaction and the fluorogenic reaction and a spectrofluorometer for the measurement of FL intensity. The most advantageous point is that the method does not require the purification of OPRT or deproteinization, which is necessary for conventional methods. The assay offers a non-laborious, low-cost, non-radioactive and facile analysis of OPRT activity in biological samples, which could not be achieved by the reported assay methods. We hope that the present method could be useful in many fields of research such as basic biochemistry, drug discovery and clinical technology.

## Data Availability

All data presented in this study are available from the corresponding author: tshibata@gunma-u.ac.jp.

## Supplementary Materials

The following supporting information can be downloaded at: https://www.mdpi.com/article/10.3390/s23052507/s1, Figure S1: Reaction specificity of the present fluorogenic reaction with 4-TFMBAO; Figure S2: Statistical analysis of OPRT reaction with different concentrations of substrate; Figure S3: Fluorescence spectra of the product of the fluorogenic reaction with 4-TFMBAO.
