# Supplementary material for: A Facile Fluorometric Assay of Orotate Phosphoribosyltransferase Activity Using a Selective Fluorogenic Reaction for Orotic Acid
Source: Sensors (Basel). 2023 Feb 24;23(5):2507. doi: 10.3390/s23052507 (PMC10007123; doi:10.3390/s23052507)
Supplement: Supplementary file 1 [file sensors-23-02507-s001.zip › sensors-2214376-supplementary.pdf]

Sensors
Supplementary Materials

A facile fluorometric assay of orotate phosphoribosyltransferase activity using a selective fluorogenic reaction for orotic acid

Takayuki Shibata*, Tomohiro Narita, Yutaka Suto, Hasina Yasmin and Tsutomu Kabashima

Table of contents

	page
Reaction specificity of the present fluorogenic reaction with 4-TFMBAO	2
Statistical analysis of OPRT reaction with different concentration of substrate	3
Fluorescence spectra of the product of the fluorogenic reaction with 4-TFMBAO	4

Reaction specificity of the present fluorogenic reaction with 4-TFMBAO

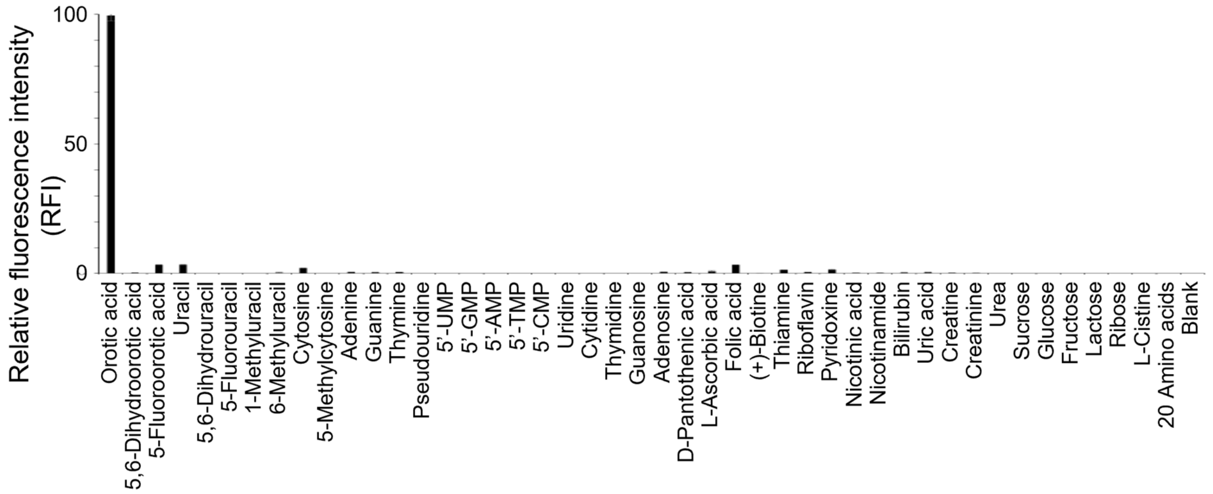

Figure S1. The reaction specificity of the present fluorogenic reaction with 4-TFMBAO to biological materials such as nucleobases, nucleotides, amino acids, nutrients and metabolites. Majority of the data were quoted from a previous report (*J. Fluorescence* **2015**, *25*, 1005-1011.).

Statistical analysis of OPRT reaction with different concentration of substrate

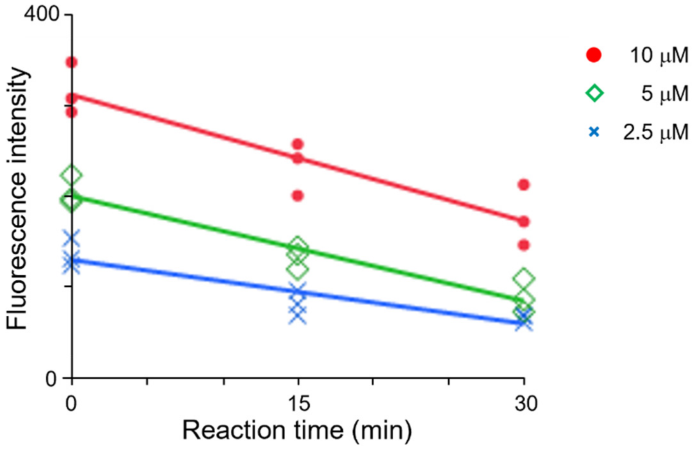

Figure S2. Regression lines between 0 to 30 min of OPRT reaction time with different concentrations of orotic acid. Statistical analysis was conducted using analysis of covariance model with the null hypothesis of no interaction between orotic acid concentration and OPRT reaction time. When parallelism of all three lines were analyzed, the resulting p-value was calculated to be 0.032. Since the null hypothesis is rejected from this result ($p < 0.05$), we can say that the alternative hypothesis, in which the three slopes are different, is correct. On the other hand, the p-value obtained with two lines, 5 mM and 10 mM orotic acid, was 0.404. It can be said that the slopes with 5 mM and 10 mM orotic acid might not be different.

Fluorescence spectra of the product of the fluorogenic reaction with 4-TFMBAO

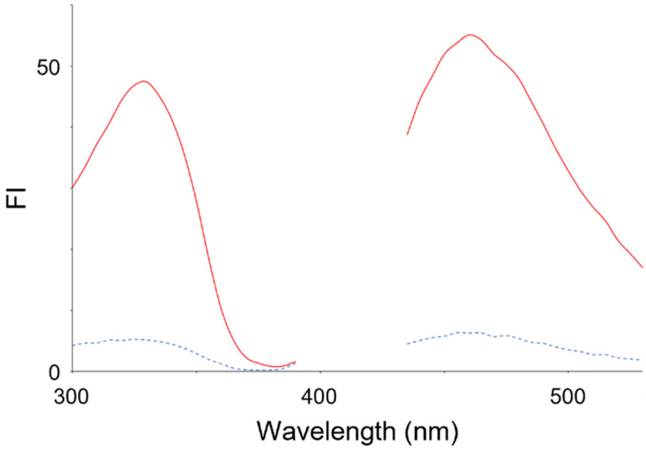

Figure S3. Emission and excitation spectra of 1 mM orotic acid (solid lines) and 10 mM 5-fluoroorotic acid (dashed lines) obtained after the fluorogenic reaction with 4-TFMBAO.
